# The Importance of a Knowledge of Bacteriology to the Dentist

**Published:** 1892-12

**Authors:** Carrie M. Stewart


### The Importance of a Knowledge of Bacteriology to the Dentist.

BY CARRIE M. STEWART, D.D.S.

The importance of a thorough knowledge of bacteriology, to the dentist, can hardly be over-estimated.

Not only does he need an acquaintance with the subject in a general way, as regards the more common germs infecting the soil, air and water, and the pathogenic germs and their action in producing disease, but he should be particularly acquainted with the germs infecting the mouth, and of these, those which may be concerned in the production of caries of the teeth, would, of course, be of interest.

Beyond the investigations of Miller, Black and a few others, little or nothing has been done in this direction by any member of the profession, and even the names of the bacteria most commonly found in the mouth are unknown to the average practitioner, although he may be comparatively well up in other branches of the profession.

It has been satisfactorily demonstrated to even the most skeptical, that bacteria do exist, and that some of them are undoubtedly the cause of certain diseases.

When a new subject is alleged to be correct in at least one part, it behooves us to satisfy ourselves by means of investigation, as to the truth or falsity of the entire subject.

The practical importance of antiseptic precautions in all operations has been shown time and again in surgery. How often could the surgeon point to a wound which had healed without the formation of pus, before the existence of the pus germ, and the proper agent for the prevention of its growth had been demonstrated, in the days before antiseptic surgery was known? Very rarely, in comparison with the large number of such cases which are constantly occurring to-day, when the value of antiseptic treatment is recognized and the action of the operator guided accordingly.

Certainly, if micro-organisms play so important a part in medicine, they are not without interest to that specialty of medicine, dentistry. Probably we would more thoroughly understand the treatment of certain forms of caries and that mysterious condition, chemical abrasion, about which nothing definite seems to be known, if we knew more about the germs of the mouth, both normally and abnormally present, and their action upon its tissues under the various influences of the body, present in health and disease.

Taking into consideration the various theories which are advanced in explanation of caries of the teeth, there is a strong probability that the bacteria theory may lead them all.

It is claimed that constitutional weakness predisposes to decay of the teeth, but it is also appreciated by the physician that constitutional weakness predisposes to any disease.

Then another theory claims a large share in the causation of caries for electrical influences. This, however, could hardly be true of the teeth alone, although the action might be indirectly most manifest upon these organs.

Comparatively great importance has been given to the acid theory.

The bacteriologist has isolated the bacilli of lactic and butyric acid, the two acids which are said to play the greatest part in the process of decay, and it has been shown that the production of these acids arises from the action of these bacilli upon disorganizing matter, and wherever these germs are present the acids are found, if the action is sufficiently prolonged. After the formation of the acid, solution of the lime salts of the teeth occurs because of the action of the acid; however, the prime cause is the bacillus of lactic or butyric acid, as the case may be.

If the teeth are not kept thoroughly clean there will be material present for the growth of bacteria, and, kept at a uniform temperature in the mouth, they flourish readily.

It cannot be denied that acid fruits, etc., will cause decay by direct action of the acid upon the tooth substance; however, as decay commonly occurs, either putrefaction or fermentation of organic matter is the first step, and neither of these processes can take place without the presence of bacteria.

Believing in this, it would seem that, as prevention is better than cure, a system of "Oral Hygiene" would be a good measure to adopt.

As to the presence of germs in the more common diseases of the mouth it is needless to treat, as they are undoubtedly present as in the diseases to which other parts of the body are liable.

Recognizing the importance of the action of germs and the harm which may result from introducing a microscopic number, consisting of thousands of individuals, into the blood, the importance of antiseptic conditions in operations upon the teeth is manifest. Not only may the gravest constitutional diseases be transmitted from patient to patient by the careless operator, but it may be that with unclean instruments, we carry the germs of caries from mouth to mouth, causing loss of teeth which might, otherwise, have been spared.

We cannot understand this subject too thoroughly, any more than we can understand the action of the bacillus tuberculosis or comma bacillus too thoroughly.

If we would keep the body in the best possible condition, we must preserve the teeth, and no mechanical contrivance will serve the purpose so well as the organs nature has designed.

To be healthy, the body must be well nourished, and to be properly nourished the nourishment must be properly prepared for assimilation, and in this preparation thorough mastication is an important factor. Anything which interferes with this, interferes with the welfare of the entire body.

If, as is probable, germs are the cause of much of the mischief occurring in the mouth, then the dentist should understand oral bacteriology, and understand it well; the dentist of the future will do so.

---

ONE of the best of Lawson Tait's axiomatic expressions is: "The road to success in the practice of our art lies not only in the knowledge how to deal with disease, but how to deal with men and women who suffer from it."

